# An autonomy-supportive climate for facilitation of self-leadership in health sciences educators

**DOI:** 10.4102/hsag.v28i0.2308

**Published:** 2023-09-29

**Authors:** Vhothusa E. Matahela, Gisela H. van Rensburg

**Affiliations:** 1 Department of Health Studies, Faculty of Human Sciences, University of South Africa, Pretoria, South Africa

**Keywords:** academic institution, autonomy-supportive climate, health sciences educators, leadership, motivation, self-leadership, self-leadership practices

## Abstract

**Background:**

The authors have observed that studies on autonomy-supportive climates in academic settings mostly focus on educator-facilitated supportive environments that motivate students towards improved performance. Yet, little is known about how academic institutions teaching nursing can facilitate autonomy-supportive climates that enhance health sciences educators’ self-leadership practices.

**Aim:**

This article discusses ‘autonomy-supportive climate’, a factor that emerged as a self-leadership practice construct, and how it can be promoted in academic institutions to facilitate self-leadership practices in health sciences educators.

**Setting:**

The study was conducted in purposively selected academic institutions (*N* = 15) located in two provinces in South Africa.

**Methods:**

Quantitative methods were employed to describe the factor ‘autonomy-supportive climate’, which yielded as a self-leadership practice construct, from a broader mixed methods project that sought to formulate guidelines that could promote health sciences educators’ self-leadership. The data were analysed using exploratory factor analysis.

**Results:**

The construct ‘autonomy-supportive climate’ is one of the five constructs in the Self-leadership Practices Subscale that was found to be meaningful and valid, with its Cronbach alpha coefficient of 0.82 being the highest in the subscale. The perceptions of participants were that an autonomy-supportive climate promotes the facilitation of the educators’ self-leadership in a nursing education setting.

**Conclusion:**

An academic institution could play a significant role in enabling self-leadership in educators, which would in turn improve their teaching performance.

**Contribution:**

This study describes autonomy-supportive climate as a facilitator of self-leadership in health sciences educators. The study’s recommendations could assist institutions in facilitating a climate that strengthens educators’ self-leadership.

## Introduction

Health sciences education in South Africa is undergoing curriculum reforms, with academic institutions required to offer new nursing curricula that are aligned with a new qualifications sub-framework. The process involves developing new curricula and investment in the learning and teaching infrastructure, ensuring that health sciences educators have the capacity to teach new programmes, and ensuring attainment of required educator to student ratios. For academic institutions, these requirements should be aligned with regulated accreditation prescripts from the Council on Higher Education and South African Nursing Council.

Central to the implementation of these reforms are educators, who are expected to be agile to function within a fast-changing and competitive climate. They engage in a myriad of teaching activities to produce nurses that are expected to contribute when addressing the health needs of the population (Department of Health 2019). Working in such a competitive climate with high job expectations and constricted timelines can bring about fierce competition and stress among educators. An academic climate characterised by fierce competition requires a teaching workforce that is self-motivated, empowered and whose autonomy is supported (Mikušová, Klabusayová & Meier 2023). The academic climate is expected to enhance educators’ motivation and engagement during teaching and learning in such a manner that the educators can provide a reciprocal milieu that offers a supportive climate to students (Kaylor & Johnson 2019). To achieve this, educators must first have higher degrees of autonomy themselves (Türk et al. 2021).

In mainstream education, it has been established that educators with high levels of autonomy tend to be efficient, experience job satisfaction and are easily retained (Salokangas, Wermke & Harvey 2020). Autonomous educators tend to perceive their academic climate in a positive manner, feel empowered by the teaching climate and are not easily burnt out compared to fellow colleagues who may have restricted autonomy. However, oftentimes educator autonomy can get eroded during implementation of reforms, especially those that involve mandatory curriculum (Ryan & Deci 2020). Likewise, institutional policies and leadership styles that are too controlling and perceived to be interfering with educators’ choices in the classroom can restrict educators’ autonomy (Ryan & Deci 2020). Typically, such reform implementation approaches would not have considered the educator’s intrinsic motivation and autonomy; as such, they will not facilitate trust and responsibility to educators and will dent their self-confidence towards the achievement of tasks and responsibilities in the institution (Bros & Schechter 2022; Ryan & Deci 2020). However, educators working in an autonomy-supportive climate feel motivated to work towards successful realisation of educational and regulatory reforms (Dixit 2022). The inclination by educators to execute educational reforms gets sustained when the teaching climate is autonomy supportive (Haug & Mork 2021).

Educator autonomy is enhanced when institutional leaders are flexible enough to allow for educators to try out new teaching strategies during delivery of pedagogy, teaching standards and assessment practices, thereby enabling educators to improve their responses and feedback to students (Ralph et al. 2020). When provided with autonomy, educators engage in self-leadership practices as they collaborate with peers, innovate and pay attention to their self-development, assisting them in overcoming the stress and emotional burdens that are associated with teaching in a rapidly changing work climate in the modern world (Ralph et al. 2020).

Self-leadership is a process through which individuals influence their thoughts, feelings and behaviours to achieve their goals (Stewart, Courtright & Manz 2019). While outdated forms of leadership emphasise on designated leaders utilising their external influences to motivate followers, self-leadership focuses on employees taking initiative to motivate themselves (Harari et al. 2021). There are three sets of self-leadership approaches, namely behaviour-focused, natural-reward and constructive thought strategies (Neck, Manz & Houghton 2020). Behaviour-focused strategies of self-observation, self-goal setting and self-reward foster feelings of self-determination and competence, helping individuals to manage their actions (Neck et al. 2020). Natural reward strategies facilitate the individual’s perceptions and creation of enjoyable features into work tasks to enhance a sense of self-control, competence and purposefulness (Harari et al. 2021). Constructive thought strategies on the other hand generate optimistic thinking habits, whereby individuals engage in positive self-talk and visualise successful performance (Harari et al. 2021).

Manz’s (1986) self-leadership theory, upon which this study draws its theoretical framework, recognises the influence of the environment on an individual’s self-leadership practice in terms of identifying intrinsically appealing aspects of the work, feelings of competence, self-control and a sense of purpose. Such an autonomy-supportive work context encourages employees to assume responsibility and accountability for their work assignments and progressively utilise self-leadership strategies to enhance own motivation and performance (Stewart et al. 2019). While self-leadership can be promoted through autonomy-supportive climates (Stewart et al. 2019), individuals must have self-leadership attributes to achieve autonomy (Hasugian, Simamora & Gaol 2021). It is when a self-leading individual works within an autonomy-supportive climate that feelings of meaning, competence and self-determination are experienced (Van Dorssen-Boog et al. 2022). According to Liao et al. (2022), self-leading individuals’ inclinations to be innovative, risk-takers and proactive are better leveraged in an autonomy-supportive climate.

Some authors have observed that self-leadership practices thrive in climates that support autonomy, thus boosting self-leadership skills and self-confidence (Nientied & Toska 2021; Ralph et al. 2020). While self-leadership is an internal process, external forces such as an autonomy-supportive climate can positively influence the practicing of self-leadership (Van Dorssen-Boog et al. 2022). This is because an autonomy-supportive climate enhances feelings of meaningfulness, competence and self-motivation to perform well in all spheres of one’s work activities, whether operational, administrative or strategic activities, leading to organisational success (Van Dorssen-Boog et al. 2022).

Given the background above, it is prudent that institutional leaders create a working climate that facilitates educators’ self-leadership through provision of an autonomy-supportive climate. Health sciences educators’ engagement in self-leadership practices can effortlessly be indoctrinated into students, subsequently producing healthcare professionals who possess self-leadership dispositions essential in yielding quality patient care. Teaching in a climate with autonomy-supportive leaders could enhance educators’ intrinsic motivation to teach, and in turn, students are more likely to be autonomously motivated to improve their performance (Ryan & Deci 2020).

While there appears to be a rich body of knowledge on the autonomy of nurses in general, the concept of health sciences educator autonomy is rarely explored. Nor does the available literature explore how academic institutions can create autonomy-supportive climates that influence health sciences educators’ self-leadership practices. Thus, the guiding question for this study was: *How can academic institutions create autonomy-supportive climates that facilitate health sciences educators’ self-leadership practices?*

The article intends to answer the research question by providing a description of the factor ‘autonomy-supportive climate’, which emerged as one of the constructs in the Self-leadership Practice Subscale in a mixed-method study that was conducted with educators teaching in purposively selected institutions in South Africa. The article further describes how an autonomy-supportive climate could be promoted in academic institutions to facilitate self-leadership practices in health sciences educators.

## Research methods and design

A descriptive quantitative design, which utilised a self-administered instrument that was based on themes from other phases (Gray & Grove 2021), was utilised to achieve the study’s aim.

### Study design

This article reports on the quantitative phase of an exploratory sequential mixed-method research project that was conducted with the aim of formulating strategies that could promote the self-leadership in health sciences educators. The broader study had three phases: an integrated literature review to explore and describe the self-leadership concept in health sciences educators, a qualitative phase wherein focus group discussions were held with a selected group of health sciences educators, and a quantitative phase that followed the first two phases. The development of the structured data collection instrument (structured questionnaire) in the quantitative phase was informed by the qualitative data. Thus, this article describes methods employed in the quantitative phase of the project. In this phase the factors related to self-leadership practices of health sciences educators were described.

### Setting, population and sample

The setting of the study was institutions located in two of the nine provinces of South Africa. The purposively selected institutions have been among the country’s largest producers of registered nurses over the past decade. The population for the quantitative phase were full-time health sciences educators who were in the employ of the institution teaching a nursing programme for a minimum of 1 year and readily available to be part of the study. For the recruitment of educators in the quantitative phase, convenience sampling was employed, ensuring that none of the respondents had previously participated in the study’s qualitative stage. Potential and willing participants who met the specified criteria were identified through research coordinators at educational institutions. Consequently, individuals who fulfilled the eligibility requirements and expressed their willingness were enlisted for the study. Participation was on a voluntary and anonymous basis, with no incentives offered. They were free to withdraw from the study at any given time without any penalty or negative effect.

### Development and pre-testing of the instrument

Development of the English-language 78-item instrument involved item generation from integrated themes of the integrated literature review and qualitative sub-phases, and self-leadership theory. The questionnaire comprised four sections: the first one elicited data on the socio-demographic characteristics of the participants, and the second section was made of 29 items which elicited the perceptions of participants of the health sciences educators’ self-leadership concept and its constructs. The third section comprised of 33 items that ascertained actual activities that could be used to describe health sciences educators’ self-leadership practices. The last section encompassed nine items that were intended to ascertain how motivation contributed to health sciences educators’ self-leadership. The authors also added explorative open-ended questions to the sections. A seven-point Likert scale that allowed for responses ranging from 1 (strongly disagree) to 7 (strongly agree) was used and pre-tested with 16 participants who could fit the criteria for inclusion but would not form part of the broader study. Pre-testing of the instrument assisted in reconsidering how questions flowed from each other, item numbering and improvement on item statement construction. The changes made to the questionnaire were only technical in nature. The findings from the pre-testing were not included in the main findings.

### Data collection

Data were collected in the years 2018 and 2019. An information leaflet describing the purpose of the study, ethical considerations in respect of participation in the study, as well as instructions on how to complete the questions, accompanied the questionnaires. The questionnaire was also distributed via the SurveyMonkey method. This was achieved through extrapolating questionnaire items into the SurveyMonkey with the assistance of the statistician. A total of 443 educators from 15 academic institutions located in the two provinces, were contacted to participate through hand-delivered instruments and SurveyMonkey. The response rate was as follows: 67% (*n* = 252) for completed questionnaires that were hand-delivered and 19% (*n* = 13) for those that were filled out using the SurveyMonkey method. Therefore, out of a possible 443 participants, 265 (59.8%) educators completed the questionnaire.

### Data analysis

Data were analysed using the descriptive Statistical Package for Social Sciences (SPSS) Version 25. Exploratory factor analysis (EFA) is the statistical technique that was employed to measure the validity of constructs, while the Cronbach alpha coefficient (α) was used to ensure the reliability of the constructs. Furthermore, the non-parametric Kruskall–Wallis test (Gray & Grove 2021) was utilised to determine if there were statistically significant differences between the mean ranks of the ‘autonomy-supportive climate’ construct and the participants’ socio-biographical properties. Findings published in this article will focus on the health sciences educators’ self-leadership practices.

### Ethical considerations

Ethical clearance was obtained from the Research Ethics Committee of the university where the study was registered (REC-012714-039). The study adhered to all the ethical requirements in accordance with the policies and procedures of the committee, and in line with the Helsinki Declaration of 1975, as revised in 2013. The authors ensured that written informed consent was obtained from all participants, and that their anonymity was preserved.

### Internal and external validity

Validity and reliability measures ensured rigour of the research. Content, construct and face validity ensured validity of the instrument. The instrument was pretested and had its items coded in efforts to improve content validity. Content validity was also ensured through the use of an integrated literature review, and deliberations between the two authors to assess whether the questions were relevant to the subject. Questionnaire pre-testing and item coding were performed to ensure instrument content validity. Face validity was ensured through reviewing the instrument content for unintended ambiguity and lack of clarity that could lead to possible misinterpretation. Construct validity was guaranteed after the authors integrated Manz’s (1986) self-leadership theory with the integrated literature review on educators’ self-leadership within the instrument items. The instrument was meticulously designed, pre-tested and reviewed to enhance reliability. According to Bell, Bryman and Harley (2019), a Cronbach’s α test is the most common method used to measure internal reliability. Thus, the authors used a Cronbach’s α of 0.6 to determine the internal consistency of the items and reliability of the instrument. Internal consistency reliability gets higher as the Cronbach’s α gets closer to 1 (Bell et al. 2019).

## Result and discussion

The good response rate (59.8%) could be attributed to the authors implementing various follow-up strategies such as phoning, emailing and revisiting academic institutions so that more educators could participate. The interest could also be borne out of curiosity as little is known of the phenomenon of self-leadership in health sciences educators (Matahela & Van Rensburg 2022).

### Socio-demographic, educational and professional profiles of the participants

Table 1 presents the participants’ socio-demographic, educational and professional profiles. A total of 265 educators with an age range of 27–72 years, average age of 49.53 years (standard deviation [SD] = 9.54 years) participated in the study. Only 15 (6%) participants were males, and 250 (94%) were females. All the educators had postgraduate qualifications, either in nursing education (*n* = 239) or nursing management (*n* = 170). In terms of teaching experience, 1 year was the lowest number, whereas the highest number was 40 years (SD = 7.76 years). In terms of the type of institution, 178 (67%) of the participants were from public colleges, 60 (23%) from private colleges, and 27 (10%) were from universities.

**TABLE 1:** Educators’ socio-demographic, educational and professional profiles (*N* = 265).

Variable	*N*	%
**Gender (*n* = 265), missing = 0**		
Female	250	94
Male	15	6
**Age (*n* = 263), missing = 2**		
≤ 40	44	16.73
41–50	79	30.04
51+	140	53.23
**Years of teaching experience (*n* = 263), missing = 2**		
1–5	69	26.2
6–10	69	26.2
11–15	72	27.4
16–20	31	11.8
21+	22	8.4
Total	263	100
**Institutional type (*n* = 265), missing = 0**		
Public college	178	67
Private college	60	23
University	27	10
Total	265	100

*Source*: Matahela, V.E., 2019, ‘Guidelines for the facilitation of self-leadership in nurse educators’, D Litt et Phil thesis, University of South Africa, Pretoria.

### Validity testing of all the self-leadership constructs through exploratory factor analysis

The EFA was performed to the responses to test validity of all the constructs in the instrument. The EFA determined if the individual instrument items loaded or contributed onto the constructs as anticipated in the instrument. Extraction of factors was done through the maximum likelihood method, which preceded the varimax rotation.

The authors’ decision on the total factors that could be used for rotation was based on cumulative percentage of variance greater than 50%, Eigen value of greater than 1.0, and a substantial decline in the scree plot. The authors used factor loading cut-off of 0.40 to interpret the loading of a factor as a reasonable item, as depicted in Table 2, which also shows the matrix of rotated factor loadings for Self-leadership Practices Subscale. Thus, the authors used bold numbers to emphasise factor loadings that satisfied the cut-off limit of 0.40 or greater on the table. Factor 1 was one of the five factors that were retained for rotation for the subscale self-leadership practices as it showed up with Eigen values that were above 1 with a cumulative variance of 50.9%. A scree plot result indicated that Factor 1 was meaningful.

**TABLE 2:** Factor loadings for the Self-leadership Practices Subscale.

Construct	Item number	Items	Factor 1	Factor 2	Factor 3	Factor 4	Factor 5
Autonomy supportive climate	C67	Health sciences educators have a responsibility to instil professional ethics and values in their students.	0.72	-	-	-	-
	C68	Leaders in an academic institution should be passionate, inspirational and build self-confidence in health sciences educators.	0.66	-	-	-	0.42
	C69	The leaders in academic institutions should give health sciences educators room for failure and encourage them to take risks.	0.43	-	-	-	-
	C70	Health sciences educators should be involved in the decision-making processes of the academic institution.	0.83	-	-	-	-
	C71	The academic institution should support health sciences educators’ innovation and creative behaviours.	0.73	-	-	-	-
	C72	Health sciences educators are change agents who advocate for the transformation of the broader community.	0.64	-	-	-	-
	C75	Academic institutions should invest in training programmes that stimulate health sciences educators’ self-leadership	0.58	-	-	-	-
Continuing professional development	C46	Individual health sciences educators should engage in their own professional development.	-	0.58	-	-	-
	C47	Health sciences educators should identify own learning needs for self-development based on the current and future health and education trends.	-	0.62	-	-	-
	C48	Health sciences educators take time to reflect on how their work contributes to the improvement of student performance.	-	0.58	-	-	-
	C50	Engagement in CPD activities that are relevant to health sciences educators’ area of work could facilitate their self-leadership.	-	0.50	-	-	-
	C54	Health sciences educators should take time to research new information and developments in their areas of teaching.	-	0.45	-	-	0.41
	C51	Health sciences educators should take time to reflect on their teaching behaviours and actions with the aim to make positive improvements and meaningful change.	-	0.57	-	-	-
	C60	Health workers are team workers who engage in sharing ideas and resources with fellow educators.	-	0.43	-	-	-
Role modelling	C56	Health sciences educators should endeavour to meet deadlines on their tasks.	-	-	0.60	-	-
	C59	Health sciences educators should give timeous feedback to the students on their performance.	-	-	0.53	-	-
	C57	Health sciences educators should adhere to teaching schedules (timetables).	-	-	0.52	-	-
	C55	Health sciences educators should strive to commence their work on time.	-	-	0.52	-	-
	C58	Health sciences educators should develop lesson plans in their preparations for teaching.	-	-	0.47	-	-
	C66	Health sciences educators should promote ethical attitudes towards colleagues, students and in society.	-	-	0.42	-	-
Shared leadership	C61	Collaboration between educators in the academic institution should be encouraged.	-	-	-	0.62	-
	C65	Inclusion of educators in succession planning ensures continuity in key leadership positions and retains intellectual and knowledge capital.	-	-	-	0.64	-
	C53	Health sciences educators should request feedback on their performance from significant others in the academic institution.	-	-	-	0.42	-
Mentoring	C63	The academic institution should provide new health sciences educators with mentors.	-	-	-	-	0.58

*Source*: Matahela, V.E., 2019, ‘Guidelines for the facilitation of self-leadership in nurse educators’, D Litt et Phil thesis, University of South Africa, Pretoria.

CPD, continuing professional development.

In order to make logical and theoretical sense, the five factors (constructs) in Table 2 were allocated names (labelled). Six items loaded on the first factor, interpreted as ‘autonomy-supportive climate’; six items loaded on the second factor, interpreted as ‘continuing professional development’; six items loaded on the third factor, interpreted as ‘role modelling’; three items loaded on the fourth factor, interpreted as ‘shared leadership’; while one item loaded on the fifth factor, which could be interpreted as ‘mentoring’.

There were two items that were found to be cross-loading. The first, item C68, cross-loaded for the first and fifth factors at 0.66 and 0.42 respectively. Thus, these items were not retained for the first and the second factors. While it loaded highest on the first factor, there was no logical nor theoretical sense to retain item C68 on the fifth factor. Likewise, the authors did not retain item C54, as it cross-loaded for both the first at 0.45 and the fifth factor at 0.40. Two items on the fifth factor, namely C68 and C54, were cross-loading with other factors, and these items were not retained. Consequently, item C63 which ascertained provision of mentors to new educators by the academic institution, was the solitary item that remained on the fifth factor. Thus, a score could not be calculated for the fifth factor as it only had one item.

### Reliability testing on self-leadership practices (Subscale C)

Subsequent to the validity testing of the constructs, evaluation of the reliability of the different constructs through item analysis was conducted using the Cronbach’s α coefficient. These tests confirmed the relevancy of construct items in measuring the construct in a reliable manner (internal consistency). Analysis results for the instrument subscale on self-leadership practices (Subscale C) are presented in Table 3. Measuring of reliability testing was done on items that loaded in the following manner: Factor 1 (six items) named ‘autonomy-supportive climate’; Factor 2 (six items) named ‘continuing professional development’; Factor 3 (six items) named ‘role modelling’ and Factor 4 (three items) named ‘shared leadership’. There was no item left out from each of the construct.

**TABLE 3:** Reliability testing on the Self-leadership Practices Subscale.

Subscale	Construct	Items	Cronbach’s α	Reliability
Self-leadership practices	Autonomy-supportive climate	C67, C69, C70, C71, C72, C75	0.82	Good
	Continuing professional development	C46, 47, 48, 50, 51, 60	0.78	Acceptable
	Role modelling	C56, 59, 57, 55, 58, 66	0.75	Acceptable
	Shared leadership	C61, 65, 53	0.69	Acceptable
	Mentoring	C63	†	†

*Source*: Matahela, V.E., 2019, ‘Guidelines for the facilitation of self-leadership in nurse educators’, D Litt et Phil thesis, University of South Africa, Pretoria.

† , Score could not be calculated.

Notably, all constructs for the subscale had a Cronbach’s α greater than 0.6, denoting reliability of the subscale constructs. Accordingly, reliability testing results denote internal consistency of the Self-leadership Subscale.

The focus of this article is on the construct ‘autonomy-supportive climate’, whose Cronbach’s α was found to be the highest in the subscale at 0.82. This indicates good reliability of the construct and suggests that its items had relatively high internal consistency. In literature, there is an explosion of interest on how leaders can provide autonomy support to employees as well as ensure sustainable organisational motivation in the climate of fierce competition (Grybauskas, Stefanini & Ghobakhloo 2022).

### The frequencies of items in the construct ‘autonomy-supportive climate’

Participants’ responses on the instrument were rated on a 7-point Likert-type scale ranging from 1 (*strongly disagree*) to 7 (strongly agree). All the items of the construct ‘autonomy supportive climate’ are now presented in terms of frequencies and percentages in Table 4. The frequencies and percentages illustrate the importance of each item as rated by the participants. Most of the items were rated high; however, the item that received the highest response is the item, namely *Health sciences educators have a responsibility to instil professional ethics and values in their students,* C67 (*n* = 211) at 79.92%, while the item that received the lowest response was item: *The leaders in academic institutions should give health sciences educators room for failure and encourage them to take risks*, C69 (*n* = 138) at 52.27%. This means that educators strongly disagreed more on item C69.

**TABLE 4:** The frequency and percentage (%) of the items in the construct ‘autonomy supportive climate’.

Item no.	1		2		3		4		5		6		7	
	*n*	%	*n*	%	*n*	%	*n*	%	*n*	%	*n*	%	*n*	%
C67	1	0.38	0	0.00	1	0.38	3	1.14	7	2.65	41	15.53	211	79.92
C69	7	2.65	0	0.00	4	1.52	16	6.06	23	8.71	76	28.79	138	52.27
C70	1	0.38	0	0.00	1	0.38	5	1.89	12	4.55	57	21.59	188	71.21
C71	0	0.00	0	0.00	2	0.76	3	1.14	12	4.55	62	23.48	185	70.08
C72	0	0.00	0	0.00	2	0.76	6	2.27	15	5.68	66	25.00	175	66.29
C75	0	0.00	1	0.38	1	0.38	8	3.03	9	3.41	49	18.56	196	74.24

### Composite score: mean and median for the construct ‘autonomy-supportive climate’

**FIGURE 1:**
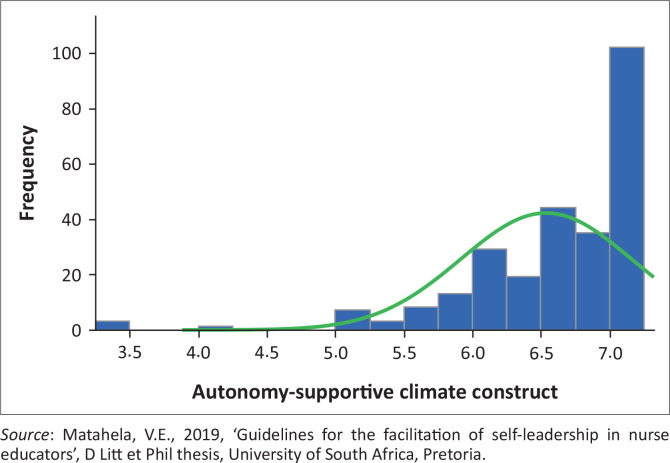
Distribution of composite scores for autonomy-supportive climate construct (*n* = 264).

Figure 1 illustrates how composite scores on the ‘autonomy-supportive climate’ construct are distributed for each participant, with instrument ratings ranging from a 1 (strongly disagree) to a 7 (strongly agree). The authors used the calculation of the average items that were deemed reliable and loaded onto the factor to determine composite construct scores of the construct ‘autonomy-supportive climate’ in the Self-leadership Practices Subscale. The authors interpreted a distribution as skew if the skewness value was outside the −1 and +1 range. In such an occurrence, the authors made use of the median to interpret and summarise the results. Each of the participants had a composite score, ranging from 3.3 to 7 (maximum score). Figure 1 depicts scores with a skewness value of −2.28 and a mean of 6.54. Consequently, the authors used the median of 6.8 for interpretation, which happens to be greater than the mean of 6.54, with a SD of 0.62. The participants’ strong agreement with the construct intimates that they perceived that health sciences educator self-leadership practices could be promoted by a climate that is autonomy-supportive.

### The effect of biographical properties on the construct ‘autonomy-supportive climate’

There seems to be a void of consensus in literature concerning whether participants’ biographical properties influence self-leadership constructs. Therefore, the authors sought to determine if educators’ gender had any statistically significant difference on the construct ‘autonomy-supportive climate’, before testing other biographical properties. Owing to the skewness of the construct ‘autonomy-supportive climate’, the nonparametric Kruskall–Wallis test was performed on the construct to establish if there were statistical differences in the construct and gender. Table 5 provides a comparison of composite scores on the gender categories for the construct ‘autonomy-supportive climate’.

**TABLE 5:** Comparison of composite scores on the gender categories for the construct ‘autonomy supportive climate’ (*n* = 264).

Gender	Frequency	Mean	Median	SD
Female	249	6.55	6.8	0.62
Male	15	6.2	6.16	0.51

Note: Kruskal–Wallis Test = χ^2^(1) = 8.8189, with the *p*-value < 0.0030.

SD, standard deviation.

The *p*-value from the Kruskall–Wallis test is less than 0.01 (*p* < 0.0030), indicating that the construct ‘autonomy-supportive climate’ is significantly different among the mean ranks of gender categories at a 95% level of confidence. The authors could not find any statistical significance for differences between the mean ranks of the construct and the participants’ remaining socio-biographical properties such as age, teaching experience, type of academic institution and educators’ additional qualifications.

## Autonomy-supportive climate as a self-leadership practice construct

‘Autonomy-supportive climate’ emerged as a construct after employment of a rigorous exploratory, descriptive sequential mixed method. The quantitative data indicates that participants perceived an autonomy-supportive climate as a facilitator of health sciences educators’ self-leadership practices. The implications of ‘autonomy-supportive climate’ emerging as a valid construct for self-leadership practices means that the construct facilitates self-leadership engagements in educators. This also implies that both the institutional leadership and to some extent the educators’ peers should strive to provide an autonomy-supportive climate that ensures that educators are provided with access expertise and resources that will support them in practising self-leadership.

As a construct, ‘autonomy-supportive climate’ in an academic institution context entailed educators perceiving that leaders in the institution provided a conducive climate that enhanced self-leadership practices through the following autonomy-related activities:

- providing educators with a supportive milieu that embraces failure and encourages taking of risks
- providing a supportive framework that includes educators in decision-making processes
- supporting educators’ engagement in self-initiated innovative and creative behaviours
- supporting educators’ empowerment in self-leadership
- empowering educators to inspire professionalism in students
- educators as change agents to the broader community.

### Providing educators with a supportive milieu that embraces failure and encourages taking of risks

Participants scored item C69 (*Leaders in academic institutions should give health sciences educators room for failure and encourage them to take risks*) lower than other items in the construct, at 52.27%. This could be due to participants perceiving that their managers and fellow colleagues may not be supportive of giving educators room for failure during learning and teaching processes. In literature, leaders in autonomy-supportive climates support employees to take risks and make mistakes in their journey towards initiative and innovative work behaviour (Bin Saeed et al. 2019). It is proposed that managers provide a climate that has a learning culture wherein educators can learn from their failures, utilise research skills to extrapolate the key lessons from mistakes in their teaching and learning activities (Hesbol 2019). Thus, academic institutions should yearn to become learning organisations that facilitate learning from failure, accept mistakes and collaboratively welcome the learning opportunities provided by failure. Such a climate would require fellow educators to view themselves as partners of co-equals who feel free to share their feelings of vulnerability to their colleagues. When there is collaborative learning, educators have a platform to provide feedback on the reasons for mistakes and failures, learn as a team, while simultaneously attaining self-leadership and a shared institutional vision (Hesbol 2019). As Klammer, Grisold and Gueldenberg (2019) assert, mistakes take individuals outside of the normal pathways (outside of the box) to allow for learning and innovative thinking.

### Inclusion of educators in decision-making processes

The participants strongly agreed with item C70 (71.21%), that ascertained whether educators believed that they should be included and actively participate in decision-making processes of the academic institution. This response could be due to participants’ understanding that freedom for independent decision-making could inspire educators to take responsibility and accountability for their decisions and actions. These are important self-leadership attributes that would keep individual educators and academic teams motivated to improve their performance. Similarly, successful organisations invite and consult subordinates to platforms where decisions are taken, listen to their thoughts and views, and incorporate their inputs into the organisation’s strategic decisions (Matahela 2019).

Aspects in which educators could seek participation, in terms of decision-making are curriculum development, aspects related to pedagogical approaches and assessments, responding to student performance and behaviour, provision of an effective teaching and learning environment, as well as professional development for lifelong learning (Eren 2020). Other examples pertain to freedom to define academic quality standards, recruitment of students into nursing programmes, leadership opportunities and ethics (Maranzan et al. 2023). However, other than the autonomy as teaching professionals in the learning and teaching processes, educators have a right to give inputs on the running of the institution and participating in academic planning, improvement, leadership and management practices (Torres, Bulkley & Kim 2020).

### Supporting educators to engage in self-initiated innovative and creative behaviours

Educators strongly agreed that the educational institution should provide support that promotes educators’ innovative and creative activities (item C71, 70.08%). Examples of innovation and creativity could be in the execution of a didactic activity, preparation of assessments, and implementation of teaching methodologies (Menezes & Novaes 2020). Although it cannot be guaranteed that innovative and creative ideas will accomplish the intended learning and teaching outcomes, the intrinsic joy, fascination and interest induced by the freedom of testing uncertainty, indistinctness and ambiguity could facilitate self-leadership in educators (Bin Saeed et al. 2019). Educators will feel safe to bring out new innovative ideas if there are collaborative relationships between supportive peers and institutional leaders who support teachers when they take risks and learn together (Dumulescu & Muţiu 2021).

### Supporting educators’ empowerment in self-leadership

Participants scored item C75 (*academic institutions should invest in training programmes that stimulate health sciences educators’ self-leadership*) second highest at 74.24%. The participants’ perception could be guided by a view that asserts that despite educators having autonomy in their own professional development, the academic institution has a role in supporting health sciences educator autonomy through supportive policies and a learning culture (King et al. 2021). Moreover, self-leadership skills training is purported to inspire self-confidence, resilience and willingness to take risks (Nientied & Toska 2021).

### Empowering educators to inspire professionalism in students

An item that was scored highest by the participants was C67 (*Health sciences educators have a responsibility to instil professional ethics and values in their students*) at 79.92%. This could mean that when provided with autonomy support, educators felt empowered with the responsibility to inculcate ethics and values in students. Educators are expected to provide a supportive learning climate that develops the students’ professional values through strengthening their capacity for ethical decision-making, thus enabling their provision of safe and ethical care (Bimray, Jooste & Julie 2019). In such a learning climate, educators promote student autonomy by passionately role modelling professional attitude and ethical sensitivity. Such teacher behaviours inspire students to develop into nurses who are highly professional, skilled and motivated to meet the population health needs (Satoh, Fujimura & Sato 2020).

### Educators as change agents to the broader community

Item C72 (*Health sciences educators are change agents who advocate for the transformation of the broader community*), was reasonably scored at 66.29%. The participants’ positive response to this item could be attributed to them assenting to the reality that educators need to be agile to adapt and respond to the ever-changing teaching climate and health developments (Klar 2020). When empowered with an autonomy-supportive climate, educators practise self-leadership to transform individuals, health care and educational systems and the society, support social justice, and contribute to the development of underserved communities (Matahela 2022). Thus, educators should in turn create an autonomy-supportive climate for students by being mindful of the disparities experienced by students on issues related to access to required learning and teaching equipment and milieu (Klar 2020).

## Significance difference between construct and categories

A non-parametric Kruskall–Wallis test revealed a significance difference between the construct scores and gender categories, with females having higher median score (6.8) than males (6.2). This means that although an autonomy-supportive climate was important for both genders, the female educators perceived an autonomy-supportive climate as more facilitative to their self-leadership practices than did the male teachers.

## Recommendations

The following recommendations for nursing education, nursing practice, policy and research are proposed.

### Recommendations for nursing education

The authors recommend that leaders in the institutions provide an autonomy-supportive work climate to educators as follows:

- Provide training to institutional managers, including heads of departments on utilisation of autonomy-supportive leadership to promote educators’ autonomy support.
- Create a climate wherein institutional leaders and managers are empowered in the promotion of educators’ reliance on intrinsic motivation as it enhances perseverance, self-efficiency and performance.
- Create a climate wherein educators feel encouraged to take initiatives and provide platforms that offer educators opportunities for making choices.
- Build a trusting relationship with educators by providing safe platforms that support educators with challenging tasks, facilitate their professional development and professional networks.
- Seek out educators’ input on policies, involve educators in decision-making processes, make attempts to appreciate issues from educators’ perspectives, and provide positive reinforcements for effective performance.
- Educators should endeavour to provide student nurses with an autonomy-supportive climate that supports students’ expression and pursuit of their personal interests and goals during the learning and teaching processes. Therefore, educators can increase students’ commitment to classroom learning and teaching activities by paying attention to students’ inner motivational resources and employing attitudes of interpersonal understanding and support.

Indeed, the interpersonal nature of providing an autonomy supportive climate means that institutional managers can be trained on how to improve their interpersonal skills that facilitate autonomy support (Mutonyi et al. 2022). Thus, institutional managers should be provided with professional training on autonomy-supportive behaviours and provisioning of an autonomy-supportive climate for health sciences educators.

### Recommendations for nursing practice

The practical nature of nursing requires that during their training, students will get placed in clinical settings for exposure to work integrated learning under the supervision and role modelling efforts of clinical preceptors. It would be beneficial to student nurses if clinical preceptors and other clinical professionals are provided with professional training on autonomy-supportive behaviours and provision of autonomy-supportive climate. This would include clinical practitioner’s effective use of their knowledge and skills, experience and clinical judgement to practise within their scopes of practice without bureaucratic restrictions, in collaboration with other health care professionals.

### Recommendations for policy

Policy makers should strive to empower the health sciences educator workforce with autonomy, self-leadership and promote autonomy-supportive climates. They should view self-leadership as an individual health sciences educator’s asset in navigating through a climate fraught with bureaucratic systems. Thus, when implementing reforms, the institution should endeavour to promote, recognise and facilitate educators’ self-leadership as a sustainable way of stimulating the educators’ intrinsic motivation and self-determination. Development of policies, rules and procedures that revolve around the influence of designated institutional leaders should be discouraged as this practice encourages dependence on external incentives for improvement of performance, which could stifle the educators’ autonomy and self-leadership.

### Recommendations for further research

The significance of autonomy in education as elucidated through the self-determination theory necessitates further description and exploration to determine specific contextual elements that characterise an autonomy-supportive climate in health sciences education settings.

## Strengths and limitations of the study

The study provides an understanding of how academic institutions could facilitate their educators’ self-leadership to improve their performance; however, some limitations are worth mentioning. One of them is that the study was conducted in academic institutions across only two of the country’s nine provinces. Another limitation is that it was conducted on one disciple of health sciences educators, namely nurse educators. As such, the present findings cannot be broadly generalised, but they can be transferrable to other academic institutions and health sciences disciplines. Lastly, another limitation to the study pertains to limited health science education literature on autonomy, to an extent that we had to borrow from the mainstream education literature, making it difficult to generalise certain information in the academic nursing context. However, the authors get solace from the understanding that the psychological determinants underlying autonomy support such as intrinsic motivation are purported to be universal (Ryan & Deci 2020). Thus, the autonomy-supportive climate construct and its effects as described in the study are relevant for diverse settings in and outside of South Africa.

## Conclusion

The article sought to describe the emergence of an autonomy-supportive climate as a self-leadership practice construct for educators using descriptive quantitative research methods. The authors propose recommendations on how an autonomy-supportive environment that facilitates self-leadership could be created by leaders in the academic institution and those in the policy-making spheres so that there could be sustainable training of health sciences students.

The country’s nursing education reforms provide an opportunity for educators and leaders in academic institutions to reflect on sustaining health sciences educator autonomy while shaping learning, teaching and leadership practices in ways that are responsive to realities and new developments that affect both the health and higher education sectors.

Institutional managers can demonstrate autonomy support by creating relationships with their educators in a manner that reinforces the health sciences educators’ perceptions that they are involved and supported in decision-making and that their managers inspire them, recognise their efforts and capabilities. Such autonomy support for health sciences educators could ultimately lead to reciprocal autonomy and improved academic performance for students. Thus, the authors further recognise the contribution made towards student training by educators working in healthcare settings (clinical practice), for example, clinical preceptors. It is recommended that they be provided with professional training on autonomy support and self-leadership to equip them with skills to create an autonomy-supportive climate for students. An example could be a safe space where growth can take place, using reflective activities to enhance learning and development.
